# A Study on the Geophylogeny of Clinical and Environmental *Vibrio cholerae* in Kenya

**DOI:** 10.1371/journal.pone.0074829

**Published:** 2013-09-16

**Authors:** John Kiiru, Ankur Mutreja, Ahmed Abade Mohamed, Racheal W. Kimani, Joyce Mwituria, Robert Onsare Sanaya, Jane Muyodi, Gunturu Revathi, Julian Parkhill, Nicholas Thomson, Gordon Dougan, Samuel Kariuki

**Affiliations:** 1 Centre for Microbiology Research, Kenya Medical Research Institute, Nairobi, Kenya; 2 Division of Microbiology, Department of Pathology, Aga Khan University Hospital, Nairobi, Kenya; 3 Centers for Disease Control and Prevention, Nairobi, Kenya; 4 Wellcome Trust Sanger Institute, Hinxton, Cambridge, United Kingdom; St. Petersburg Pasteur Institute, Russian Federation

## Abstract

Cholera remains a significant public health challenge in many sub-Saharan countries including Kenya. We have performed a combination of phylogenetic and phenotypic analysis based on whole genome DNA sequences derived from 40 environmental and 57 clinical *V. cholerae* from different regions of Kenya isolated between 2005 and 2010. Some environmental and all clinical isolates mapped back onto wave three of the monophyletic seventh pandemic *V. cholerae* El Tor phylogeny but other environmental isolates were phylogenetically very distinct. Thus, the genomes of the Kenyan *V. cholerae* O1 El Tor isolates are clonally related to other El Tor *V. cholerae* isolated elsewhere in the world and similarly harbour antibiotic resistance-associated STX elements. Further, the Kenyan O1 El Tor isolates fall into two distinct clades that may have entered Kenya independently.

## Introduction

Cholera, caused by the bacterium *Vibrio cholerae*, is still a common disease in many parts of the world where public health infrastructure is compromised. Significantly, although the Bay of Bengal is traditionally associated with the origin of pandemic cholera, 66% of the all cases reported between 1995 and 2005 were in sub-Saharan countries (http://www.who.int/cholera/statistics/en) [1]. In Kenya, the first official case of cholera was reported in 1971 [2] and since then at least 18 discrete outbreaks have been documented [3-8]. For instance from 2000 to 2006, the number of cases notified to the World Health Organisation (WHO) each year ranged from 816 to 1,157. In 2007, a cumulative total of 625 cases resulting in 35 deaths were reported in four regions; Rift Valley (West Pokot, Turkana), Coast (Kwale), North Eastern (Garissa, Wajir, Mandera) and Nyanza (Kisumu, Bondo and Siaya). In addition from January–April 2008, in the Lake Victoria region (Suba, Migori, Homabay, Rongo, Siaya, Kisumu, Bondo, Nyando, Kisii South), outbreaks resulted in 790 cases and 53 deaths. During the period January 2009-May 2010, cholera was reported in other regions including the coast with a total of 11,769 cases and 274 deaths (http://www.who.int/wer/2012/wer8731_32.pdf) [7,8]. To date, little is known about how the cholera cases in these different regions are related phylogenetically.

Links between cholera and rainfall, sea surface temperature and plankton have been reported in regions where this disease is considered endemic [4,9-12], but the emergence and spread of cholera within local and national boundaries is not fully understood. Of the 200 serogroups of *V. cholerae* only O1 and O139 are associated with epidemic disease [13]. O1 isolates can be assigned to two biotypes, known as classical and El Tor, the latter being responsible for the current global seventh pandemic [14]. El Tor isolates form a relatively conserved monophyletic lineage that emerges periodically from foci in the Bay of Bengal with the potential to spread globally and some Kenyan *V. cholerae* El Tor isolates have previously been mapped back onto this phylogeny [14].

Recent work on Kenyan cholera isolates identified 5 Multi-Locus Variant Analysis (MLVA) clonal complexes circulating in Kenya [15]. However, MLVA does not provide a phylogenetic context and therefore has limited utility in the tracking of outbreaks. In this study, we have applied whole genome sequencing linked to phylogenetic and phenotypic analysis to characterize both environmental and clinical *V. cholerae* in Kenya, providing a countrywide analysis of the phylo-dynamics of this disease.

## Methods

### Isolation and characterisation of *Vibrio cholerae*

Clinical isolates were collected from distinct outbreaks that occurred in different parts of Kenya from 2005 to 2010 (Figure S1). The outbreak affected regions were: Busia along the Ugandan border (elevation 1500 meters, average annual rainfall 2000 mm); Kisumu on the shore of Lake Victoria (elevation 1200 meters, average annual rainfall 2000 mm); Malindi, Kilifi and Kwale along the Kenya’s Indian Ocean coast (elevation 20 m, average annual rainfall 1050 mm); Nairobi, an area with high population density of approximately 150,000 and poor sanitation in the Eastland informal settlements (15 km east of the city, elevation 1800 m, average annual rainfall 750 mm); Thika town 50 km north of Nairobi (elevation 1800, average annual rainfall 800 mm); Kakuma near Lake Turkana; West Pokot and Daadab refugee camps and the adjacent town of Garissa in northeast Kenya (elevation 0-250 m, annual rainfall less than 250 mm). In most parts of Kenya, January-February are dry months, followed by a rainy season from March-June, the exception for the sites we sampled being Kakuma, West Pokot and Daadab, which are semi-arid all year-round. For consistency, a distinct outbreak was defined as a gap of at least 2 months between the last known cholera case and a report of a new case in the same location. Archived isolates were initially subcultured on thiosulphate citrate bile salts sucrose agar (TCBS) and confirmation of isolate identity was undertaken by serology using polyvalent, anti-Ogawa, and anti-Inaba antisera (Denka Seiken, Tokyo, Japan). To test for haemolysis *V. cholerae* were grown on 5% sheep blood nutrient agar plates incubated at 37°C overnight. The presence of *V. cholerae* virulence genes specific for classical and El Tor (ctxAB, *ompU, hylA, toxR, zot, tcpA*) was analysed using whole genome sequencing [16].

Environmental *Vibrio cholerae* were isolated from water, plant materials and sediments from unprotected boreholes, wells, permanent and seasonal rivers, ponds and surface runoffs using methods described by Huq et al. [17]. Along Lake Victoria, water samples were collected at least 5-10 m from the shoreline. Similarly, ocean water, plant materials and sediments were collected along bays, estuaries and beaches along the Kenyan coastline and from shallow oceanic shelves of the Indian Ocean. In each sampling site, preference was given to watering points and places with evidence of human activity such as washing, bathing, fish landing and sale points. All samples were transported to the laboratory in an insulated cool box to maintain a temperature close to that of the water at each collection site and processed within 24h of collection. Environmental samples were cultured as follows: 10 ml of each plant homogenate and sediment sample was enriched in 5 ml triple strength alkaline peptone water and incubated for 18 h. Subculture was performed from alkaline peptone water onto TCBS agar and incubated for 18 h. Yellow mucoid colonies suspected to be *V. cholerae* were then further defined using biochemical tests and confirmed by serology.

These field studies did not involve endangered or protected species from the sampling sites. No vertebrate animals were sampled. For sampling materials from individual homes permission and consent was sought from each owner and we administered a short questionnaire to obtain information on use of the water in the home. For sampling along the rivers, ponds and surface runoff there was no need for any approvals as we did not sample any plant or animal species. To sample along Lake Victoria shores and Indian ocean shores, we sought approval from fisheries department and had technicians from the department involved in helping to obtain samples in the respective sites.

#### Antimicrobial susceptibility testing

Antimicrobial susceptibility tests were performed using commercial discs following manufacturer’s instructions (Oxoid, Basingstoke, UK). Antimicrobials tested included ampicillin (10 µg), cefuroxime (30ug), ciprofloxacin (5 µg) and nalidixic acid (30 µg) which were used for testing susceptibility to the quinolones. Aminoglycosides used in susceptibility tests included kanamycin (30 µg), streptomycin (30 µg), and gentamicin (10 µg). We also tested tetracycline (30 µg), chloramphenicol (30 µg), furazolidone (50 µg), sulphamethoxazole (25 µg) and trimethoprim (5.2 µg). *Escherichia coli* ATCC 25922 was used as a control for bacterial growth and potency of antibiotic discs. Susceptibility tests were interpreted using the Clinical and Laboratory Standards Institute guidelines (2012).

### Genome sequencing

Multiplex sequencing libraries of 250 bp insertion size were created and loaded on the Illumina HiSeq cell to perform 72-base paired-end sequencing of 96 separate libraries in each lane. Each library had a unique index tag and after sequencing this tag sequence information was used for assigning reads to the individual samples. All the samples achieved an average coverage of 200x in the regions where SNPs were called. All the data has been submitted to European Nucleotide Archive with the accession codes listed in Tables S1 and S2.

### Whole genome alignment and detection of SNPs

The 72-base paired-end read data obtained was mapped to the O1 El Tor reference N16961 (NCBI accession numbers AE003852 and AE003853) using SMALT (http://www.sanger.ac.uk/resources/software/smalt) to obtain a whole genome alignment for all the strains in this study. For SNP calling, the approach of Harris et al. [18] was used. No SNPs were called from the reads that either did not map to N16961 or from the regions that were absent from the N16961 reference genome. Strict filtering of the SNPs was performed and any SNP with a quality score less than 30 were excluded. Also, a SNP was considered true only if it was present in at least 75% of the reads at any heterogeneously mapped ambiguous sites. High-density SNP clusters and the possible recombination sites were excluded using he methodology previously described [19].

### Phylogenetic Analysis

Default settings of RAxML v0.7.4 [20] were used to estimate the phylogenetic trees based on all the SNPs recorded against the genome as explained above. The number of SNPs on each branch were calculated by reconstructing all the polymorphic events on the tree using PAML [21]. M66 (accession numbers CP001233 and CP001234), a pre-seventh pandemic strain and a well-known out-group for the seventh pandemic strains, was used to root the final phylogenetic tree [14]. For visualization and ordering of the nodes, phylogenetic tree reading software Fig tree (http://tree.bio.ed.ac.uk/software/figtree/) was used.

### Comparative Genomics

A multi-contig draft genome was generated for each sample by assembling the paired end reads using a *de-novo* genome assembly program Velvet v0.7.03 [22]. The parameters were set to give the best kmer size and at least 20X kmer coverage. Contigs were ordered using Abacas as per the reference N16961 El Tor complete genome sequence [23,24]. Annotation was transferred from the reference sequence to each ordered draft assembly. Artemis Comparison Tool was used for manual comparison of the assembled genomes [25].

### Linear Regression Analysis

The final phylogenetic tree was opened using Path-O-Gen v1.3 (http://tree.bio.ed.ac.uk/software/pathogen) and the root-to-tip distance data for each strain was exported to excel. This data was used to plot a linear regression curve against the year of isolation of the strain. The R-squared correlation, slope and p-values were determined using the inbuilt regression package of R statistical environment.

### Bayesian Analysis

The tree was reconstructed and the ancestral or nodal dates for the Kenyan clade and sub-clades were inferred using the Bayesian Markov Chain Monte Carlo framework [26]. The final SNP alignment without recombinant sites was used as the input dataset for BEAST [26] and the rates of evolution on the branches of the tree were estimated using a relaxed molecular clock [27], providing the flexibility for the rates of evolution to change amongst the branches of the tree. A coalescent constant population size and a GTR model with gamma correction was used and the results produced from three independent chains of 100 million steps each sampled every 10,000 steps to maintain homogeneity. The first 10 million steps of each chain were binned. The results of the three chains were combined using Log Combiner, and the maximum clade credibility tree was generated using Tree Annotator software in the BEAST package (http://tree.bio.ed.ac.uk/software/beast/). ESS cut off value of 200 was used for each parameter and convergence was visually confirmed using Tracer 1.5 (http://tree.bio.ed.ac.uk/software/tracer).

#### Ethics Statement

The protocol for this study was approved by the Kenya Medical Research Institution Review Board reference SSC No. 1110 for both clinical and environmental field and laboratory studies. Relevant permissions and consent was sought for all field studies including environmental sampling.

## Results and Discussion

### Properties of clinical and environmental *V. cholerae*

In order to undertake a whole genome based phylogenetic analysis of *V. cholerae* from Kenya we assembled a collection of clinical and environmental isolates from different regions of the country (see Methods for full details of isolation and phenotypic procedures). In total 57 clinical *V. cholerae* isolates were obtained from cases of cholera, and 40 isolates were collected from environmental sources. The environmental isolates were derived from nine study sites and from water ranging from pH 4 to 9.7. The demography of sample sites ranged from waters with algal blooms to areas where regular household and farming activities on going (Methods and Table S2). Water samples, plant materials and sediments from unprotected boreholes, wells, rivers, and surface runoffs were collected in the following towns bordering Kenya’s Indian Ocean coast: Mombasa, Malindi, Kilifi and Kwale. Four sites were sampled along the shore of Lake Victoria (Kisumu, Siaya, Homa Bay and Kendu Bay) and in Western Kenya the three district towns of Busia, Vihiga and Kakamega were also sampled (Tables S1 and S2).

### Serotype and antimicrobial susceptibility testing of Kenyan isolates

All the Kenyan O1 *V. cholerae* isolates, whether clinical or environmentally sourced, were found to subtype serologically as Inaba, whereas some of the environmental isolates were non-O1. All clinical isolates were resistant to multiple antibiotics, including nalidixic acid, trimethoprim, sulphamethoxazole, streptomycin and furazolidone. In contrast, 66% of the environmental isolates were resistant to sulphamethoxazole, 15% to furazolidone, 56% to ampicillin and 5% to trimethoprim. Noticeably, all the clinical isolates were fully susceptible to ampicillin. In addition both clinical and environmental *V. cholerae* isolates were also fully susceptible to tetracycline, cefuroxime, chloramphenicol and ciprofloxacin (Table S1 and Table S2). This contrasts to some extent with previous Kenyan studies [28] in which 8% and 3% of clinical and environmental isolates, respectively, from around Lake Victoria were resistant to tetracycline. This resistance trend has some similarities to the picture emerging in endemic areas of Bangladesh where isolates are now uniformly resistant to trimethoprim/sulfamethoxazole and furazolidone and resistance to tetracycline and erythromycin shows temporal fluctuation with variations from year to year [29]. *V. cholerae* O1 resistant to tetracycline have previously been reported in Zambia [28] in the 1990s, but those isolated from Ethiopia [5] and Somalia [30] in the same period were susceptible to this antibiotic.

### Whole genome sequencing and phylogenetic analysis

**Figure 1:**
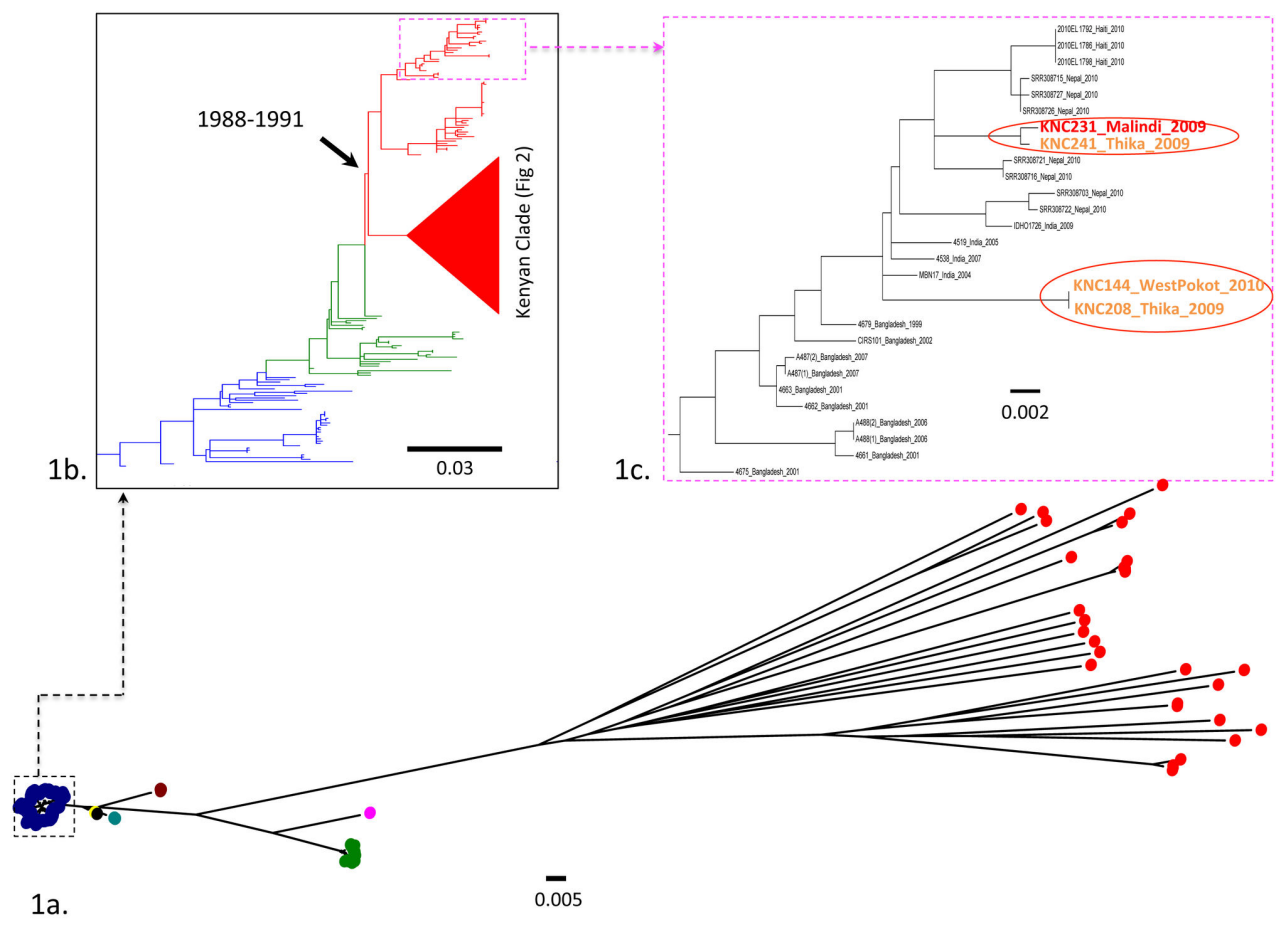
Global and seventh pandemic phylogeny. 1a, a maximum likelihood phylogenetic tree of *V*. *cholerae* based on the SNP differences within the core genome. The 6 major O1 clinical groups are shown in this tree with the 7^th^ pandemic El Tor in blue, classical lineage in green and other colours are match the colours of strains in Table S2. In red are the environmental non O1/O139 strains from Kenya. The date range on the wave 3 node is the BEAST estimated time when the seventh pandemic wave 3 cholera entered Kenya. 1b, a maximum likelihood phylogenetic tree of the 7th pandemic lineage of *V*. *cholerae* based on the SNP differences across the whole core genome, excluding likely recombination events. The pre-7th pandemic isolate M66 was used as an outgroup to root the tree. Blue, green and red branches and the clade cartoon represent wave 1, 2, 3 and Kenyan clade respectively. 1c, a maximum likelihood phylogenetic sub-tree showing the position of Kenyan sporadic or travel linked clustering with south Asian strains. All the scales are given as the number of substitutions per variable site.

DNA prepared from 57 clinical and 40 environmental isolates (Table S1 and Table S2) were sequenced and the data generated was compared to previously published *V. cholerae* sequences taken from Mutreja et al. [14] and Hendriksen et al. [31]. The initial consensus phylogenetic tree generated from this data (Figure 1a) showed that 27 of the environmental isolates clustered well outside of the seventh pandemic lineage and differed by more than 50,000 SNPs from the reference. Further, only 49% to 89% of their sequence reads mapped onto the *V. cholerae* El Tor reference genome N16961. Thus, such isolates are clearly distinct from *V. cholerae* O1 El Tor. However, significantly, the remaining 13 environmentally sourced isolates clustered with other O1 El Tor seventh pandemic isolates and these were also serotypically O1 positive. Additionally, 98% of their sequence reads mapped onto the N16961 El Tor reference genome, differing by only ~250 SNPs from the reference sequence. The genomes of these isolates also harboured key signature genomic loci including VSP-1 and 2 and the SXT multiple antibiotic resistance associated loci found within wave 3 seventh pandemic isolates [14]. This data unequivocally confirms that these *V. cholerae O1* isolates are members of the seventh pandemic *V. cholerae* El Tor phylogeny as part of wave three described in Mutreja et al. [14].

The presence of environmental *V. cholerae* isolates phylogenetically distinct from the main El Tor lineage is important. Such isolates may be associated with diarrhoeal diseases distinct from cholera in the respective local communities and it would be worth investigating this possibility further. Additionally, such isolates clearly exist in similar environments to clinically important *V. cholerae* El Tor, presenting the possibility of genetic recombination and the exchange of antibiotic resistance determinants between these phylogenetically distinct populations.

To gain a more detailed understanding of the location of the El Tor isolates within the El Tor seventh pandemic lineage we constructed a further genome-wide SNP based phylogenetic tree (Figure 1b) by filtering out high density SNPs and removing any variation that could be a consequence of recombination using the method of Croucher et al. [19]. This tree was based on 1828 variable sites. Here, 53 O1 serogroup Kenyan isolates clustered within the wave three of the global seventh pandemic lineage (Figure 1b), with 49 isolates forming an exclusive Kenyan clade alongside 17 previously published Kenyan isolates within the wave three lineage (Figure 1b). Interestingly, four *V. cholerae* O1 isolates (KNC231, KNC241, KNC144 and KNC208) clustered in distinct positions within a clade of isolates from South Asia (Figure 1c), showing that they occupy distinct positions within the wave three lineages that could have been brought into Kenya independently, possibly by travellers.

**Figure 2:**
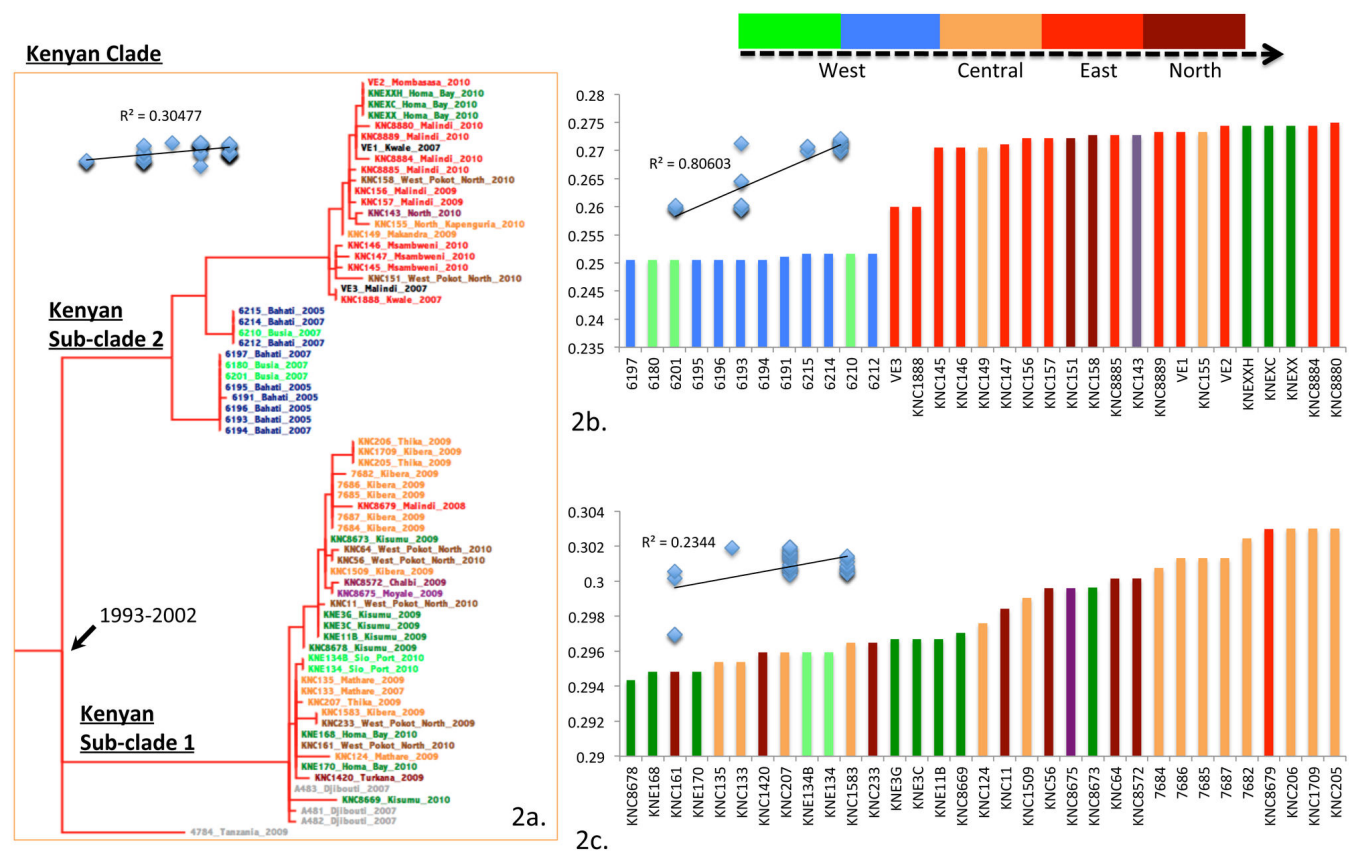
Phylogeny of Kenyan strains showing the two subclades.

To gain further insight into the temporal and spatial distribution of the Kenyan lineages we reconstructed the tree and dated all the nodes using Bayesian analysis, which is a tool for mapping isolates against time. In the temporal-spatial analysis we were also able to estimate that wave three of the seventh pandemic entered Kenya around 1988-1991, a refinement on our previous estimate using fewer Kenyan isolates (1989-1997) [14]. We then performed a linear regression analysis on the Kenyan clade by plotting the root-to-tip distance of each isolate against time of isolation, and this data showed some consistency with our previous findings that Kenyan cholera isolates are evolving in a clock-like manner (Figure 2a, 2b and 2c). By refining this analysis we were able to further subdivide the dominant Kenyan clade into two sub-clades, designated KSC1 and KSC2 (Figure 2a). Most of the isolates collected between 2005 and 2010 cluster within one of these two sub-clades. The most recent common ancestor for these two sub-clades was estimated to have emerged between 1993-2002 (Figure 2a).

2a, sub tree from the 7^th^ pandemic maximum likelihood phylogenetic tree showing the Kenyan clade and its two sub-clades. The nodal date represents tMRCA of the two sub-clades. 2b, c show root to tip distances of strains of sub-clades KSC2 and KSC1 arranged in increasing order of magnitude. The R^2^ values and the linear regression curves are based on root to tip distance *vs.* time (years) on vertical and horizontal axes respectively. The colours of the strains in 2a and bars in 2b, c represent the locations where the sample was collected. The root to tip distance for strains from Djibouti and Tanzania in KSC1 are not provided in 2c.We found some evidence of regional clustering for clinical isolates within sub-clades KSC1 and KSC2 (Figure 2b and 2c). For example, with few exceptions, isolates from the Nairobi region fell within the KSC1 sub-clade while most isolates (clinical and O1-positive environmental) from the Indian Ocean coast region (Mombasa, Msambweni, Kwale and Malindi) fell within KSC2, as did those from Busia on the Kenya-Uganda border. Other isolates from the Lake Victoria region of Kisumu and Sio-Port clustered in KSC1 whilst isolates from the Homa-Bay area were distributed in both KSC1 and KSC2. The isolates from the semi-arid region of West Pokot in Northern Kenya were also distributed in both the sub-clades, as were those from environmental sources near Lake Victoria (Figure 2b and c). There was a strong correlation between root-to-tip distance and time for KSC2 (R^2^ = 0.8). The phylo-geographic analysis of KSC2 is consistent with the notion that cholera may emerge from in and around Lake Victoria and spread to the central and eastern parts of Kenya (Figure 2b). However, the same correlation for KSC1 was weak (R^2^ = 0.2) and phylo-geographically inconclusive. Currently, we do not know how cholera entered Kenya but the fact that we were able to identify two sub-clades and some possible travel linked outliers on the *V. cholerae* El Tor tree indicates that there may have been multiple and perhaps on going introductions of this disease into the country. Further epidemiological and phylogenetic analysis will be required to establish any of these possibilities. However, we know that cholera has the potential to spread globally and jump from region to region. Comparisons with similar phylogenetic studies reported in other regions will be useful in defining how the disease is evolving [2,30-32].

Using this geophylogenetic analysis we are also able to speculate about how cholera is spreading and persisting within Kenya as our approaches are applicable to any potential outbreak or region and have a more general utility. For example, the geographical and temporal distribution of isolates of clade KSC-2 shows correlation with the previous proposals that busy highway connecting the Indian Ocean coastal towns to the Lake Victoria region plays an important role in transmission during epidemic outbreaks [8].

### Assembly and detailed comparison of genomes

For each isolate we performed *de novo* assemblies on the short read data and manually compared these assemblies against the N16961 reference genome. We also catalogued all the genomic islands found in the Kenyan isolates (see Table S3). All of the Kenyan O1 El Tor isolates possessed the *Vibrio*-associated Seventh Pandemic Islands known as VSP-1 and 2 and members of the R391 family ICE/SXT multiple antibiotic resistance cassettes. With three exceptions, all non-O1 isolates lacked classic virulence related elements such as VPI-1, VPI-2, VSP-1, VSP-2 and CTX. The three exceptions were KNE056B_2, KNE17 and KNE150. KNE056B_2 and KNE17 possessed CTX and VPI-1, whereas KNE056B_2 also possessed VPI-2. Of note, KNE150 carried R391-ICE inserted into the peptide release chain factor-3 gene (*prf*C-3), the site specific for the insertion of SXT [32]. Uniquely, every isolate in the Kenyan clades KSC-1 and KSC-2 harboured a 4 gene (VC0495-VC0498) deletion in the VSP-2 island. Also, consistent with previous findings, the small number of Kenyan wave three isolates that did not cluster within the exclusive Kenyan clade possessed an 18 gene (VC0495-VC0516) deletion characteristic of the South Asian isolates with which they clustered.

All the Kenyan isolates that mapped within wave three of the *V. cholerae* El Tor lineage harboured the R391-ICE/SXT element associated with antibiotic resistance, correlating with their resistance phenotype. This is consistent with data obtained from analysis of *V. cholerae* outbreak isolates from a previous study [33]. Clearly our data shows that antibiotic resistance is phenotypically expressed in both the environmental and clinical *V. cholerae* isolates. Interestingly, with the exception of two isolates, the other Kenyan clinical samples have an identical antibiotic resistance profile whereas the samples collected from the environmental sources had varied resistance profiles irrespective of where they clustered in the phylogenetic tree. We do know that many of these resistance determinants are associated with the STX elements and that these are hot spots for recombinogenic activity within *V. cholerae*, likely providing a mechanism for more rapid evolution of resistance.

When we analysed the *ctx*B gene type for each sequenced isolate, with the exception of KNE231 and KNE241 that harboured the *ctx*B-3b gene [14], all other Kenyan O1 El Tor isolates harboured the *ctx*B-3 toxin allele [14]. Interestingly, the non-O1 environmental isolates, KNE056B_2 and KNE17, harboured an identical *ctx*B gene, which displayed 14 SNP differences to the reference El Tor *ctx*B gene. To our knowledge this is the first time a *ctx*B gene with this sequence type has been identified in non-O1/O139 *V. cholerae*. Database searches revealed that the closest match to this novel *ctx*B gene was found in a non-O1 environmental isolate J31W reported from Argentina in 2009 (Genbank accession FJ748608), which differs by a single base pair to these Kenyan isolates at position 282. The *ctx*B gene of J31W, in contrast, has the same sequence as the El Tor reference N16961 *ctx*B at this position. The alignment showing the *ctx*B genes of N16961, KNE056B_2, KNE17 and J31W is shown in Figure S2.

## Conclusion

We have used a combination of whole genome sequencing, phylogenetic analysis and phenotyping to define a unique set of *V. cholerae* circulating in the environment or causing clinical cholera in Kenya. Kenya can arguably be regarded as an exemplar country for the sub-Saharan region and a model for their association with cholera. Our data clearly shows that many environmental *V. cholerae* isolates are phylogenetically distinct from the monophyletic seventh pandemic lineage of *V. cholerae* El Tor. However, we identified multiple *V. cholerae* O1 isolates from environmental samples that firmly mapped onto the *V. cholerae* El Tor seventh pandemic lineage, indicating potential contamination of aquatic habitats by human activities. Importantly, this suggests that the levels of environmental contamination by potentially pathogenic *V. cholerae* O1 El Tor are significant and a likely source of human infection. We were able to assign the Kenyan lineages into two sub-clades that may have entered the region independently. The clade specific signatures and the phylogenetic position of the O1 El Tor isolates outside the exclusive Kenyan clade strengthen the travel and transmission link of these cases to South Asia. Hence, it would be worth following the future outbreaks to determine if these sporadic isolates establish themselves in Kenya and elicit new outbreaks. Finally, we observed a correlation of antibiotic resistance pattern and source of sample suggesting that the pool of resistance determinants circulating within the phylogenetically distinct environment *V. cholerae* and those of the El Tor lineage in Kenya may be evolving at least in part independently. However, as such determinants predominantly reside within mobile STX type elements, which are hyper-recombinogenic, there is potential for genetic exchange between these reservoirs.

## Supporting Information

Figure S1 **Map of Kenya showing sites as red dots where the study isolates of *V. cholerae* were obtained.**
(TIF)

Figure S2 **Clustal X 2.1 multiple nucleotide sequence alignment showing the *ctxB* sequences of KNE17, KNE056B, J31W and N16961 aligned using clustal X.** The base positions with * indicate a match and those with a gap indicate a mismatch.
(TIF)

Table S1 **Clinical isolates and their antimicrobial susceptibility patterns.**
(XLSX)

Table S2 **Environmental isolates and their antimicrobial susceptibility patterns.**
(XLSX)

Table S3 **Non-O1/O139 Kenyan Environmental isolates and the genomic islands found in this study.**
(XLSX)
