# Supplementary figures and images for: A Study on the Geophylogeny of Clinical and Environmental Vibrio cholerae in Kenya
Source: PLoS One. 2013 Sep 16;8(9):e74829. doi: 10.1371/journal.pone.0074829 (PMC3774669; doi:10.1371/journal.pone.0074829)

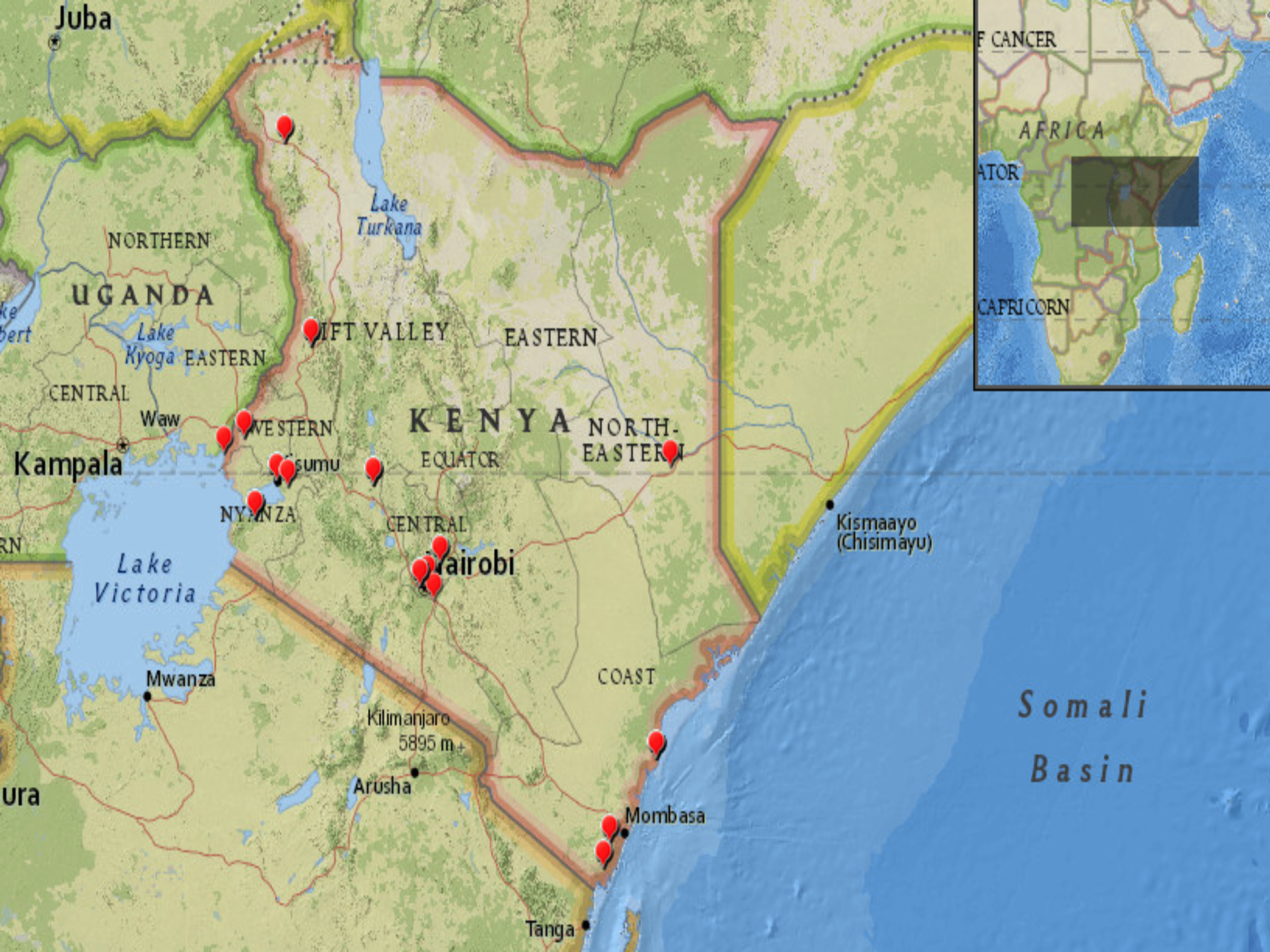

Supplement: Figure S1 — Map of Kenya showing sites as red dots where the study isolates of V. cholerae were obtained. (TIF) [file pone.0074829.s001.tif]

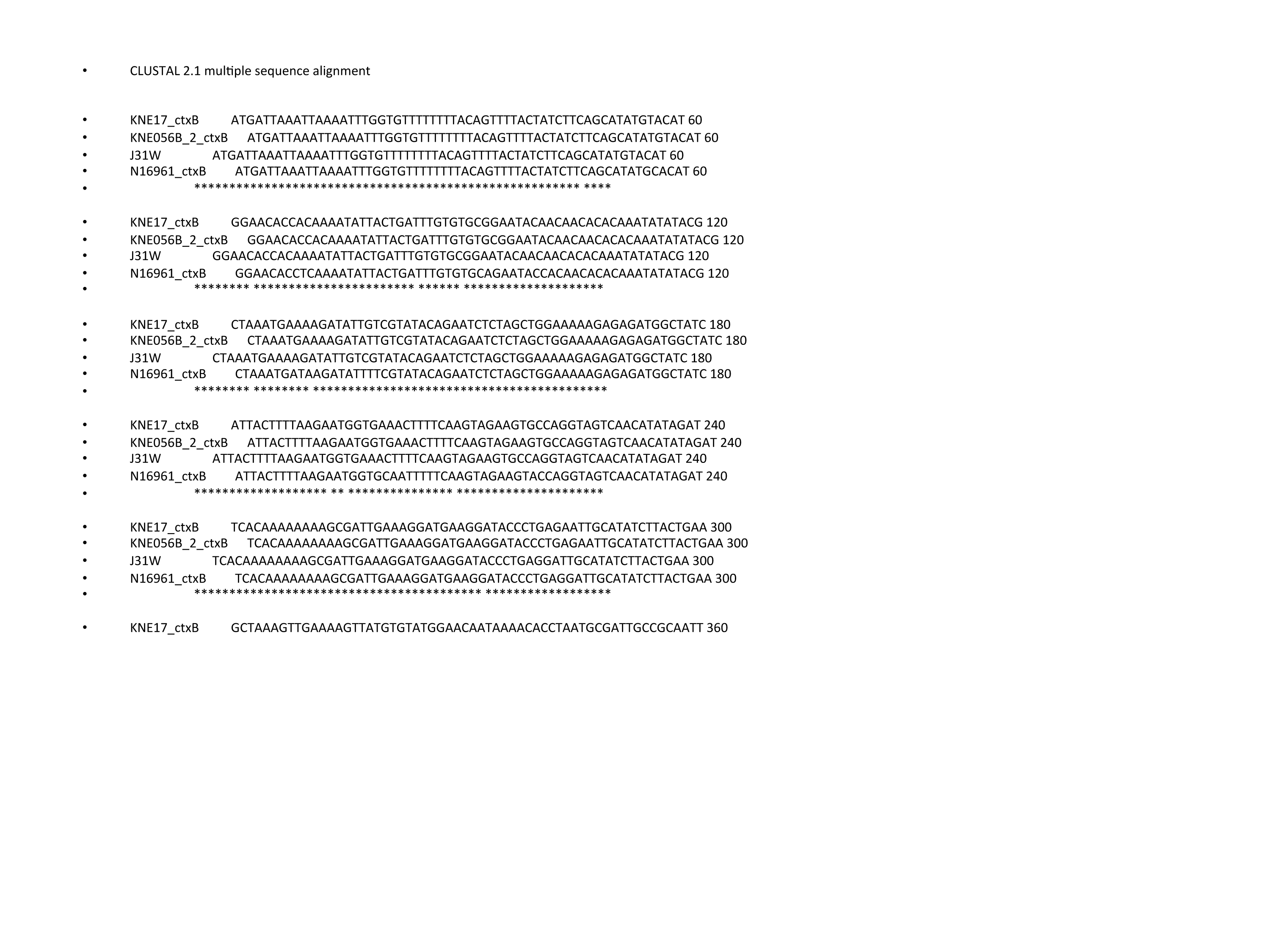

Supplement: Figure S2 — Clustal X 2.1 multiple nucleotide sequence alignment showing the ctxB sequences of KNE17, KNE056B, J31W and N16961 aligned using clustal X. The base positions with * indicate a match and those with a gap indicate a mismatch. (TIF) [file pone.0074829.s002.tif]
